# Career perspectives for young cardiologists in the Netherlands: an update

**DOI:** 10.1007/s12471-023-01816-w

**Published:** 2023-09-14

**Authors:** Lena Bosch, Madelon Minneboo, Vivan J. M. Baggen, Joost C. Beusekamp, Dilek Yilmaz, Dany Haroun, Veerle M. M. Vorselaars, Wouter C. Meijers

**Affiliations:** 1 Department of Cardiology, University Medical Centre Utrecht, Utrecht, The Netherlands; 2 Department of Cardiology, Amsterdam University Medical Centre, location Academic Medical Centre, Amsterdam, The Netherlands; 3 Department of Cardiology, Erasmus Medical Centre Rotterdam, Rotterdam, The Netherlands; 4 Department of Cardiology, University Medical Centre Groningen, Groningen, The Netherlands; 5 Department of Cardiology, Leiden University Medical Centre, Leiden, The Netherlands; 6 Department of Cardiology, Medisch Spectrum Twente, Enschede, The Netherlands; 7 Department of Cardiology, St. Antonius Hospital, Nieuwegein, The Netherlands

Dear Editor,

The article by Vorselaars et al. on career perspectives for young cardiologists in the Netherlands, published in a recent issue of the *Netherlands Heart Journal*, demonstrated low unemployment rates but only reflects the situation up to 2020 [1]. The Dutch labour market fluctuates, and the main issue for young cardiologists is the increased and uncertain time to a permanent position. The Junior Board (*De Juniorkamer*) of the Netherlands Society of Cardiology (*Nederlandse Vereniging voor Cardiologie*) actively monitors the Dutch labour market. Here we present a brief update on the current situation.

We conducted a new digital survey among 329 young cardiologists (response rate 81%, *n* = 266) who completed their training between 2016 and 2021. The unemployment rate remains low (0.4%), yet temporary positions remain frequent. Three years after registration, 34% had not gained a permanent position. However, comparing the two cohorts [1], the temporary position rate decreased slightly: from 41 to 34%. Furthermore, a smaller group (68%) of young cardiologists described the current job market as problematic, compared to 77% in the previous cohort.

These new insights highlight the fact that temporary positions remain common practice in the Dutch labour market. This might cause a heavy burden on young cardiologists, due to financial and geographical insecurity often during an important and busy phase in their personal lives. These temporary positions are accompanied by less autonomy and decreased control over the work environment that may consequently lead to a decline in job satisfaction and increased risk of burnout. Also, continuity of patient care is impaired due to alternating young cardiologists in temporary positions. This brief update demonstrates a gradual decline in temporary positions, but they remain a long-term issue for many. This is reflected by the persistently high number of young cardiologists that consider the current job market problematic (68%). As mentioned by Vorselaars et al., the high number of temporary positions is not limited to cardiology and has been observed in many medical specialties in the Netherlands [1]. This phenomenon might reflect the uncertain financial situation of many Dutch hospitals due to the limitation of financial growth for hospital care, as permitted by the Dutch government (*Het Hoofdlijnenakkoord*). As Junior Board, we will continue to monitor the job market for young cardiologists in the Netherlands. We hope the current trend towards more permanent positions continues, since it is important for job satisfaction and motivation among young cardiologists. Furthermore, it will aid in recruiting medical students and residents to initiate the training necessary to become a cardiologist.
